# Medication prescribing in face-to-face versus telehealth consultations during the COVID-19 pandemic in Australian general practice: a retrospective observational study

**DOI:** 10.3399/BJGPO.2021.0132

**Published:** 2022-01-26

**Authors:** Nasir Wabe, Judith Thomas, Gorkem Sezgin, Muhammad Kashif Sheikh, Emma Gault, Andrew Georgiou

**Affiliations:** 1 Centre for Health Systems and Safety Research, Australian Institute of Health Innovation, Macquarie University, North Ryde, Australia; 2 Electronic Medical Record Subject Matter Expert, ICT West Gippsland Healthcare Group, Warragul, Australia; 3 Digital Health and ICT, Gippsland Primary Health Network, Traralgon, Australia; 4 Gippsland Primary Health Network, Bairnsdale, Australia

**Keywords:** general practice, COVID-19, telemedicine

## Abstract

**Background:**

There has been a precipitous rise in telehealth use in general practice during the COVID-19 pandemic. Understanding differences between face-to-face and telehealth consulting is an important component for planning the future use of telehealth services beyond the pandemic. However, there is limited evidence on whether telehealth consulting impacts medication prescribing under pandemic circumstances.

**Aim:**

To compare medication prescribing in face-to-face consultations with telehealth during the COVID-19 pandemic in Australian general practice.

**Design & setting:**

A multisite, retrospective observational study. De-identified routinely collected electronic health data were used, which were extracted from 806 general practices in Victoria and New South Wales (NSW), Australia, between April and December 2020.

**Method:**

The primary outcome measure was whether at least one medication was prescribed following a telehealth or face-to-face consultation. Data were reported by medication and for each of the Anatomical Therapeutic Chemical (ATC) classification system level 1 groups. The secondary outcome measure was first-time prescribing. Telehealth included both telephone and video consultations.

**Results:**

A total of 13 608 216 consultations satisfied the inclusion criteria (61.0% face to face and 39.0% telehealth). Most telehealth consultations were conducted via telephone (97.8%). Overall, 39.3% of face-to-face and 33.0% of telehealth consultations prescribed at least one medication, which is a statistically significant difference (adjusted odds ratio [OR] 1.38, 95% confidence interval [CI] = 1.379 to 1.381). The prescribing rate was greater for face-to-face versus telehealth consultations for all drug groups except ATC level 1N (nervous system).

**Conclusion:**

Under COVID-19 restrictions in the states of Victoria and NSW, Australia, medication prescribing was higher in face-to-face consultations when compared with telehealth consultations in the study population.

## How this fits in

The COVID-19 pandemic has triggered a dramatic increase in the use of telehealth modalities for consulting in general practice. There is a paucity of quantitative evidence regarding differences between face-to-face and telehealth consultations during the pandemic, particularly for medication prescribing. This large multisite observational study of 806 general practices in two Australian states found a statistically significant difference in medication prescribing between face-to-face and telehealth consultations, with 6.3% more prescriptions issued in face-to-face consultations. Future qualitative research could explore GP decision-making criteria for prescribing medications during telehealth consultations.

## Introduction

Since the World Health Organization (WHO) declared COVID-19 (the disease caused by severe acute respiratory syndrome coronavirus 2 [SARS-CoV-2]) as a pandemic on 11 March 2020, within 15 months (11 June 2021) there were more than 174 million confirmed cases of COVID-19 and the tragic loss of more than 3.7 million lives^1^ globally. The pandemic has had a devastating impact on healthcare systems around the world, with many countries propelled into adopting changes to meet emerging challenges and increasing system demands. One measure widely introduced, or upscaled, during the pandemic was the use of telehealth modalities (including both telephone and video consultations)^2–4^ to address the challenges associated with COVID-19 such as infection control, physical distancing, and COVID-safe restricted activity directives.

Telehealth has been defined by the International Organization for Standardization as *‘the use of telecommunications techniques for the purpose of providing telemedicine, medical education and health education over distance* *‘*,^5^ and telehealth consultation uptake has rapidly accelerated worldwide.^6^ In general practice, primary care, and outpatient settings studies have reported decreases in the overall number of patient consultations,^3,7–9^ decreases in face-to-face consultations,^3,7–11^ and concomitant increases in telehealth and remote consultations,^3,4,8–13^ with telephone consults more widely used than video.^3,4,8,10,13^ While the expeditious shift towards telehealth consultations has provided accessibility to healthcare practitioners during periods of lockdown and restrictions, differences between the content of US primary care telemedicine and face-to-face consultations (for blood pressure and cholesterol assessments) during the pandemic have been reported.^11^

Another aspect of primary care consulting that has the potential to differ between telehealth and face to face is medication prescribing. A pre-pandemic systematic review of primary care antibiotic prescribing via remote consultations found mixed evidence of the impact of remote consulting with studies reporting higher, lower, and no difference in antibiotic prescribing between face-to-face and remote consultations.^14^ During the pandemic, several studies have examined antibiotic prescribing in primary care;^9,15–17^ however, fewer studies have examined the relationship between telehealth and prescribing. Researchers from the US found similar rates of new medication prescribing during the second quarter of 2020 between face-to-face and telemedicine visits;^11^ and lower rates of antibiotic prescribing for acute rhinosinusitis for face-to-face consultations (March–May 2019) compared with ‘virtual visits‘ (March–May 2020).^18^

With the precipitous rise in telehealth consultations during the pandemic, gaining an understanding of its impact on prescribing in primary care is an important component for planning the future use of telehealth beyond the current COVID-19 pandemic. Building on the authors’ previous research, which compared socioeconomic and demographic factors in the uptake of telehealth^19^ and changes in medication prescribing during the pandemic in Australian general practice,^20^ the purpose of the current study was to compare medication prescribing in face-to-face consultations with telehealth consultations during the COVID-19 pandemic in Australian general practice.

## Method

### Study design and setting

A multisite, retrospective observational study was conducted utilising routinely collected electronic health data, which were extracted from 806 general practices across five primary health networks (PHNs) in the states of Victoria and NSW, Australia. Three of the PHNs were from Victoria, including two in metropolitan Melbourne and one in a mainly rural area. The two other PHNs were from NSW, including one in metropolitan Sydney and one incorporating both metropolitan and rural areas of NSW. The study period was from 1 April 2020–31 December 2020.

### Study context

As part of the Australian Government’s response to the pandemic, a primary care package was introduced, which included temporary Medicare Benefits Schedule (MBS) items for telehealth from 13 March 2020.^21^ Temporary telehealth service items could be billed by GPs and other medical practitioners for non-hospital patients with whom they had an ‘established clinical relationship‘ and included separate item numbers for telehealth via video and telephone consultation.^22^ In addition, the COVID-19 National Health Plan announced that electronic prescribing (e-Prescribing) and dispensing would be fast tracked over a period of 8 weeks.^23,24^ The e-Prescribing provisions included options for prescriptions to be faxed or emailed to a patient’s preferred pharmacy or sent directly to the patient via short message service (SMS) or email.^24^ Further contextual information is presented in Supplementary Figure S1.

### Participants

**Figure 1.**
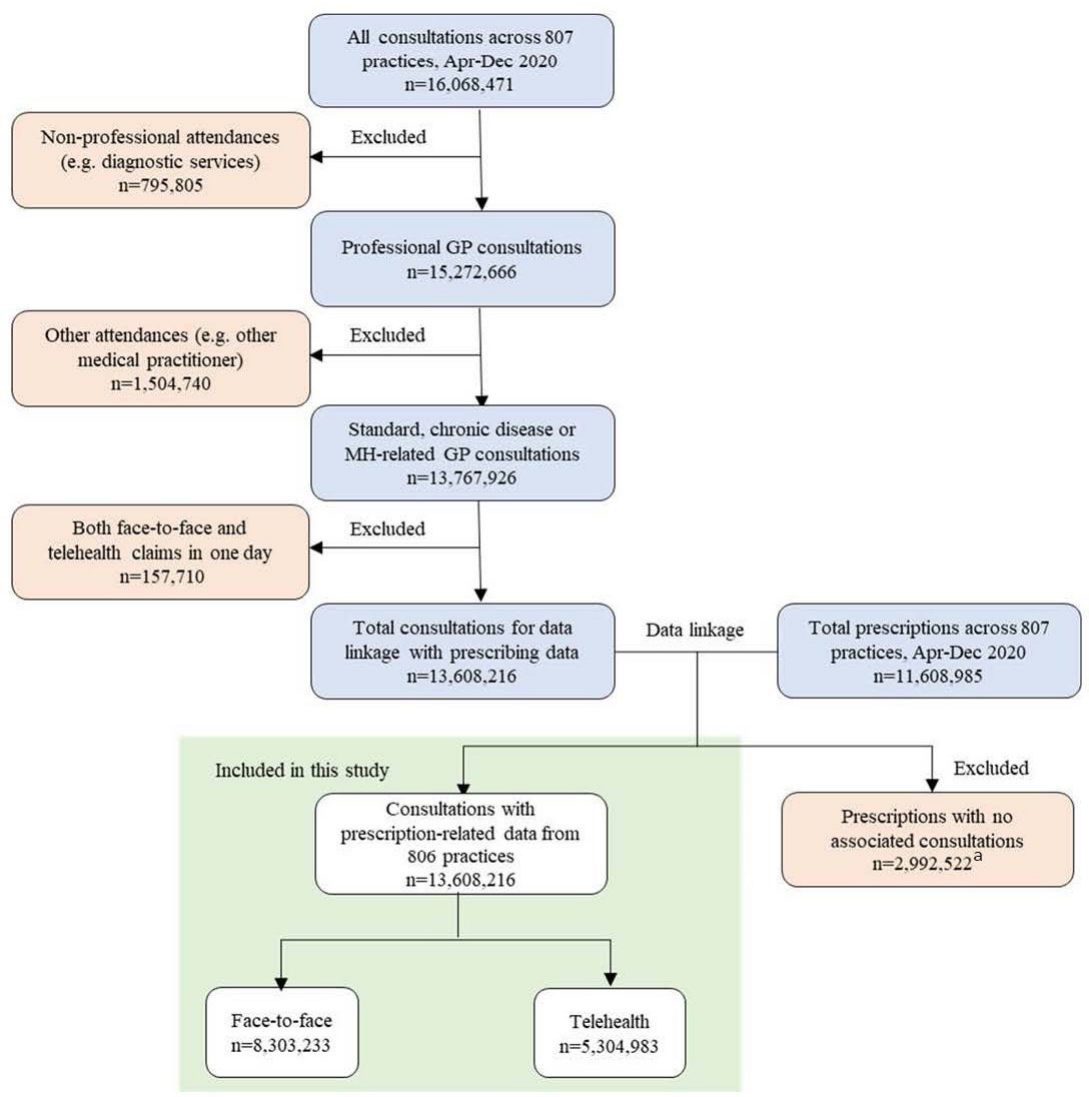
Participant selection flow chart, April–December 2020. ^a^Indicates prescriptions not associated (linked) with the selected Medicare Benefit Scheme items for standard, chronic disease management, and mental health-related consultations. MH = mental health.

The study participants included patients who received professional GP consultations for standard attendance, chronic disease management, and/or mental health services during the study period (Figure 1). The MBS items used to identify these services are presented in Supplementary Table S1. Patients seeking other services (for example, diagnostic testing) and non-GP consultations (for example, other medical practitioners, specialists, consultant physicians, psychiatrists, nurse practitioners, and other allied health practitioners attendances) were excluded. Patients who received both face-to-face and telehealth consultations in 1 day were also excluded, as the aim of the study was to compare the two consultation types in terms of medication prescribing.

### Data source

De-identified general practice data sourced from the Population Level Analysis and Reporting (POLAR) platform were utilised. The platform covers nearly 30% of the Australian population from over 800 general practices across Victoria and NSW.^17^ The details about POLAR platform have been published elsewhere.^25,26^ In brief, some of the key data collected included patient demographics (age, sex, patient postcode, and patient status), service provided (MBS items, service group [for example, standard attendance or chronic disease management], and service date), and medication prescribed (name, dose, quantity, strength, frequency, repeats, prescribing date, and the ATC codes of the medication).

The patient status (active or non-active) was determined using the Royal Australian College of General Practitioners (RACGP) criteria, whereby a patient is considered ‘active’ if they have attended the practice or service ≥3 times in the past 2 years.^27^ The patient’s postcode was used to determine remoteness of residential location and socioeconomic status of the patients based on the Australian Accessibility/Remoteness Index and the Index of Relative Socioeconomic Advantage and Disadvantage (IRSAD).^28^ The ATC system is developed by the WHO to classify active ingredients of medications based on their site of action (organ or system) and therapeutic, pharmacological, and chemical properties.^29^ According to the system, medications are classified in a hierarchy with five different levels, with each level having a certain number of groups or subgroups. For instance, ATC level 1 has 14 main anatomical or pharmacological groups and level 3 has over 260 chemical, pharmacological, or therapeutic subgroups.^29^

### Variables

The primary outcome measure was whether at least one medication was prescribed following the consultation. Results are presented for any medication prescribing; and separately for each of the ATC level 1 groups. The secondary outcome measure was first-time prescribing. The patient’s medication prescribing history was reviewed over the past 4 years (since January 2017) to determine whether a given medication was prescribed for the first time or not. The primary independent variable was the consultation type (face to face versus telehealth). Telehealth included both telephone and video consultations. The covariates considered in the model included sex, age (grouped as <40, 40–59, 60–74, and ≥75 years), socioeconomic status, patient status, remoteness index, PHN, and the state of the practice.

### Statistical methods

Descriptive statistics, including medians (interquartile range [IQR]) and frequency (%), were reported. Multilevel mixed-effects logistic models with practice-specific random intercepts were used to evaluate the association between the consultation type (face to face versus telehealth) and outcome measures (medication prescribing). This approach adjusts for the clustering effect while also controlling for known confounders. The analyses were adjusted for both patient case-mix (sex, age, socioeconomic status, patient status, and the remoteness index) and practice characteristics (PHN and the state of the practice). The strength of association was estimated using the OR with 95% CIs.

**Figure 2.**
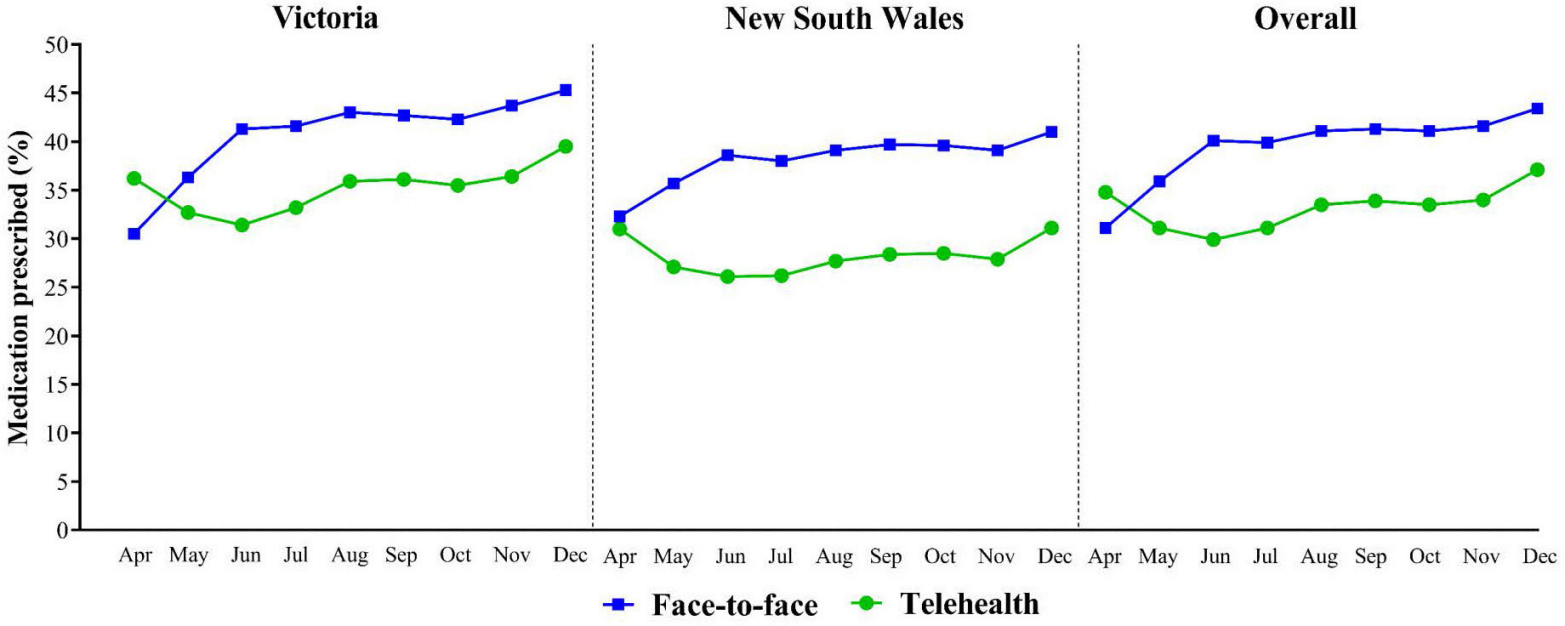
Risk-adjusted rate of prescribing at least one medication by month of GP consultation, April–December 2020. Risk-adjustment included patient level variables (age, sex, socioeconomic status, patient status, and the remoteness index)

The risk-adjusted medication prescribing rate (Figure 2) was determined using logistic regression with a robust variance estimation using a similar method as described previously by Lenzi *et al*.^30^ Patient baseline characteristics including age, sex, socioeconomic status, patient status, and the remoteness index were used to generate the risk-adjusted rate. All *P*-values were 2-tailed and *P*<0.05 was considered statistically significant. Analyses were conducted using Stata (version 16).

## Results

### Participants

A total of 13 608 216 patient consultations (8 235 130 from Victoria and 5 373 086 from NSW) for either standard, chronic disease management, or mental health from 806 practices satisfied the inclusion criteria. Of these, 61.0% (*n* = 8 303 233) were face-to-face consultations and 39.0% (*n* = 5 304 983) were telehealth consultations (Figure 1). The majority of telehealth consultations involved telephone (97.8%, *n* = 5 188 643). Table 1 presents the demographic characteristics of consultations.

**Table 1.** Baseline characteristics of consultations, April–December 2020

**Variables, *n* (%**)	**Face to face**(***n* = 8 303 233**)	**Telehealth**(***n* = 5 304 983**)	**Overall (*n* = 13 608 216**)
Sex, Female	4 684 918 (56.4)	3 324 348 (62.7)	8 009 266 (58.9)
**Age group** **, years**			
<40	3 342 444 (40.3)	2 086 727 (39.3)	5 429 171 (39.9)
40–59	2 063 824 (24.9)	1 420 669 (26.8)	3 484 493 (25.6)
60–74	1 714 115 (20.6)	1 030 080 (19.4)	2 744 195 (20.2)
≥75	1 182 850 (14.2)	767 507 (14.5)	1 950 357 (14.3)
**Socioeconomic status/IRSAD** ^**a**^			
1 (most disadvantaged)	932 407 (11.3)	429 285 (8.1)	1 361 692 (10.0)
2	1 089 856 (13.2)	559 819 (10.6)	1 649 675 (12.2)
3	1 101 025 (13.3)	795 165 (15.0)	1 896 190 (14.0)
4	1 933 384 (23.3)	1 292 530 (24.4)	3 225 914 (23.8)
5 (most advantaged)	3 227 349 (39.0)	2 216 393 (41.7)	5 443 742 (40.1)
**Remoteness** ^**a**^			
Major cities	7 417 738 (89.5)	4 695 032 (88.7)	12 112 770 (89.2)
Inner regional	793 798 (9.6)	560 362 (10.6)	1 354 160 (10.0)
Outer regional or remote or very remote	73 194 (0.9)	38 290 (0.7)	111 484 (0.8)
**Patient status**			
Active	7 646 925 (92.1)	5 116 207 (96.4)	12 763 132 (93.8)
Non-active	656 308 (7.9)	188 776 (3.6)	845 084 (6.2)
**State**			
Victoria	4 470 203 (53.8)	3 764 927 (71.0)	8 235 130 (60.5)
NSW	3 833 030 (46.2)	1 540 056 (29.0)	5 373 086 (39.5)

^a^≈0.2% missing data. IRSAD = Index of Relative Socioeconomic Advantage and Disadvantage. NSW = New South Wales.

### Medication prescribing patterns

The proportion of consultations with at least one medication prescribed was 36.9% (*n* = 5 016 626), regardless of consultation type. There was a total of 8 616 463 prescriptions. Medication prescribing patterns, according to the ATC levels 1 and 3, are shown in Supplementary Table S2. Medications for the nervous system followed by the cardiovascular system were the leading ATC level 1 drug classes accounting for 24.9% and 18.2% of all prescriptions, respectively. The top 15 ATC level 3 drug classes accounted for 53.7% of the total prescriptions. ‘Opioids’, ‘antidepressants’, and ‘lipid-modifying agents, plain’ were the top three ATC level 3 drug classes accounting for 6.5%, 5.3%, and 5.2% of all prescriptions, respectively (see Supplementary Table S2).

### Primary outcome: differences in medication prescribing

Overall, 39.3% (*n* = 3 264 748) of face-to-face and 33.0% (*n* = 1 751 878) of telehealth consultations prescribed at least one medication, which is a statistically significant difference of 6.3% (adjusted OR 1.38, 95% CI = 1.379 to 1.381). The difference in prescribing rate between face-to-face and telehealth consultations was greater in NSW compared with Victoria (9.9% versus 5.4%) (Table 2).

**Table 2.** Difference in medication prescribing between face-to-face and telehealth consultations, April–December 2020

**Consultation type**		**Number of consultations with at least one medication prescribed, *n* (%**)	**Total consultations, *n***	**Face to face versus telehealth**	
				**Difference, %**	**OR (95% CI)^a^**
Victoria	Face to face	1 807 097 (40.4)	4 470 203	5.4	1.28 (1.270 to 1.281)
	Telehealth	1 319 473 (35.0)	3 764 927		
NSW	Face to face	1 457 651 (38.0)	3 833 030	9.9	1.62 (1.619 to 1.630)
	Telehealth	432 405 (28.1)	1 540 056		
Overall	Face to face	3 264 748 (39.3)	8 303 233	6.3	1.38 (1.379 to 1.381)
	Telehealth	1 751 878 (33.0)	5 304 983		

^a^Adjusted for age, sex, socioeconomic status, patient status, remoteness, primary health network, and the state of the practice. NSW = New South Wales.

The difference in prescribing between face-to-face and telehealth consultations for each of the ATC level 1 drug groups is shown in Supplementary Figures S2–S3. The prescribing rate was greater for face-to-face versus telehealth consultations for all drug groups except for ATC level 1N (nervous system). The highest difference in prescribing rate between face-to-face and telehealth consultations was observed for ATC level J (anti-infective for systemic use) prescribing (8.2% versus 5.5%, a difference of 2.7%). However, ATC code D (dermatologicals) had the highest adjusted OR of 2.67 (95% CI = 2.64 to 2.69).

Figure 2 presents the adjusted medication prescribing rate for face-to-face versus telehealth consultations over time. The prescribing rate was greater for telehealth consultations in April (34.8% versus 31.1%), but from May onwards, the rate was consistently greater for face-to-face consultations. The peak difference in the prescribing rate between the two consultation types was reached in June with a difference of 10.2% (9.9% in Victoria and 12.5% in NSW).

### Secondary outcome: differences in first-time prescribing

Of the total 5 016 626 consultations with prescriptions, 41.0% (*n* = 2 057 545) were prescribed a medication for the first time. Overall, the proportion of consultations with first-time prescription was 18.3% (*n* = 1 520 401) for face-to-face and 10.1% (*n* = 537 144) for telehealth consultations, which is a difference of 8.2% (adjusted OR 2.03, 95% CI = 2.020 to 2.031). The difference was slightly higher for NSW compared with Victoria (Table 3).

**Table 3.** Difference in first-time medication prescribing between face-to-face and telehealth consultations, April–December 2020

**Consultation type**		**Number of consultations with a first-time prescription,** ***n* (%**)	**Total consultations, *n***	**Face to face versus telehealth**	
				**Difference, %**	**OR (95% CI)^a^**
Victoria	Face to face	830 677 (18.6)	4 470 203	7.9	1.93 (1.929 to 1.940)
	Telehealth	401 378 (10.7)	3 764 927		
NSW	Face to face	689 724 (18.0)	3 833 030	9.2	2.33 (2.320 to 2.350)
	Telehealth	135 766 (8.8)	1 540 056		
Overall	Face to face	1 520 401 (18.3)	8 303 233	8.2	2.03 (2.020 to 2.031)
	Telehealth	537 144 (10.1)	5 304 983		

^a^Adjusted for age, sex, socioeconomic status, patient status, remoteness, PHN, and the state of the practice. NSW = New South Wales.

## Discussion

### Summary

In this multisite, retrospective observational study of general practices using routinely collected health data from over 13.6 million patient consultations, face-to-face consultations were more prevalent than those conducted via telehealth (61.0% versus 39.0%). Telehealth consultations were predominantly telephone (97.8%). Overall, there was a statistically significant higher (+6.3%) prescribing rate for face-to-face consultations. During the study period, April–December 2020, the prescribing rate was only greater for telehealth consultations in April towards the end of the first wave of the pandemic in Australia,^31^ but from May onwards the rate was consistently greater for face-to-face consultations, despite a second wave in Victoria between late June and early September.^31^ Prescribing rate was greater for face-to-face versus telehealth consultations for all ATC groups, except for medications for nervous system. The proportion of consultations with a first-time prescription was 8.2% higher in face-to-face consultations.

### Strengths and limitations

A major strength of the current study is the population sample size of over 13.6 million patient consultations. The large sample size also permitted direct comparison of face-to-face and telehealth consultations during the same time period. In addition, the study period extends beyond the early phase of the pandemic and captures two distinct waves encompassing the middle of the first wave in both NSW and Victoria, and the second wave in Victoria alone (late June to early September in 2020). However, it did not capture the beginning of the first wave of the pandemic when there may have been increases in medication prescribing^32^ owing to consumer stockpiling. The study is limited to the context of general practice in two Australian states within a national COVID-19 policy environment and may not be generalisable to other settings or beyond pandemic circumstances. The analysis did not determine whether a telehealth consultation resulted in a follow-up face-to-face consultation for the purpose of medication prescribing. The analyses were performed using routinely collected data and may therefore be subject to unmeasured confounding factors.

### Comparison with existing literature

During the study period, telehealth represented 39.0% of general practice consultations. In contrast, a study by Murphy *et al* in UK primary care found 90% of GP consultations during April 2020 were conducted via remote consultation;^3^ however, this may reflect contextual differences in case numbers, local restrictions, and a shorter study timeframe compared with the current study. Murphy *et al* ^3^ also reported greater use of telephone compared with video consultations, which is consistent with the present study findings and pandemic-period reports of Veterans Affairs outpatients in the US^8^ and general practice in Australia.^13^

The need to acknowledge context (COVID-19 restrictions and policies) and timeframe (waves of pandemic) in comparing literature during the pandemic is highlighted in the present study findings where prescribing rate was greater for telehealth consultations in April (towards the end of the initial wave of the pandemic in Australia), but from May onwards the rate was consistently higher for face-to-face consultations (despite a second wave in Victoria). The April prescribing data resembles data from a study of primary health care in Iceland between March and April 2020, which showed an increase in the number of prescriptions issued from web- and telephone-based consultations, with overall numbers for web- and telephone-issued prescriptions being higher when compared with office and home visits^33^ (although relatively higher numbers were also apparent pre-pandemic).^33^

Overall, the risk-adjusted prescribing rate in the study was higher for face-to-face consultations post-April 2020, with the prescribing rate greater for all drug groups except for medications for nervous system. The study fills the knowledge gap for medication prescribing between telehealth and face-to-face consultations across all medication categories, as there is currently little comparable evidence examining the relationship between general practice consultation modality and medication prescribing during the pandemic, with prescribing studies focusing on antibiotics^18^ and/or pre-pandemic periods.^14,34^ In a study of antibiotic prescribing for acute rhinosinusitis, Miller *et al* reported a significantly lower rate of antibiotic prescribing in virtual patient visits during March–May 2020 when compared with in-person visits during the same months of the previous year.^18^ New medication prescribing in US primary care was studied by Alexander *et al* who found similar proportions between telemedicine (39.3%) and office-based consultations (44.9%) during quarter two of 2020.^11^ This is slightly less than the results from the present study period, which found the proportion of consultations with a first-time prescription was 8.2% higher in face-to-face when compared with telehealth consultations.

### Implications for research and practice

Understanding differences between face-to-face and telehealth consulting is important for planning the future use of telehealth modalities beyond the pandemic. The study findings for medication prescribing add valuable evidence to the limited literature currently available for general practice prescribing by mode of consultation during pandemic circumstances. The significantly higher prescribing rate for face-to-face consultations found in the current study suggests that prescribing may be an important factor in choice of consultation modality, particularly for new medication prescribing. As detailed by Greenhalgh and Rosen,^35^ choice of consultation modality is impacted by a complex interaction of many contributing factors. Future qualitative research could explore GP decision-making criteria for determining whether medications are prescribed during telehealth consultations.
